# Viscoelasticity measured by shear wave elastography in a rat model of nonalcoholic fatty liver disease: comparison with dynamic mechanical analysis

**DOI:** 10.1186/s12938-021-00879-3

**Published:** 2021-05-03

**Authors:** Zhaoke Pi, Mengke Wang, Haoming Lin, Yanrong Guo, Siping Chen, Xianfen Diao, Hui Xia, Guoqiang Liu, Jie Zeng, Xinyu Zhang, Xin Chen

**Affiliations:** 1 School of Biomedical Engineering, Health Science Center, Shenzhen University, National-Regional Key Technology Engineering Laboratory for Medical Ultrasound, Guangdong Key Laboratory for Biomedical Measurements and Ultrasound Imaging, Shenzhen, 518000 China; 2 Institute of Electrical Engineering, Chinese Academy of Sciences, Beijing, 100000 China; 3 Department of Medical Ultrasonics, Third Affiliated Hospital of Sun Yat-Sen University, Guangzhou, 510000 China

**Keywords:** Nonalcoholic fatty liver disease, Shear wave elastography, Dynamic mechanical analysis, Viscoelasticity

## Abstract

**Background:**

Nonalcoholic fatty liver disease (NAFLD) is rapidly becoming one of the most common liver diseases. Ultrasound elastography has been used for the diagnosis of NAFLD. However, clinical research on steatosis by elastography technology has mainly focused on steatosis with fibrosis or non-alcoholic steatohepatitis (NASH), while steatosis without fibrosis has been poorly studied. Moreover, the relationship between liver viscoelasticity and steatosis grade is not clear. In this study, we evaluated the degree of liver steatosis in a simple steatosis rat model using shear wave elastography (SWE).

**Results:**

The viscoelasticity values of 69 rats with hepatic steatosis were measured quantitatively by SWE in vivo and validated by a dynamic mechanical analysis (DMA) test. Pathological sections were used to determine the steatosis grade for each rat. The results showed that the elasticity values *µ* obtained by the two methods followed the same trend, and *µ* is significantly correlated with liver steatosis. The Pearson’s correlation coefficients indicate that $\mu$ obtained by SWE is positively linear correlated with DMA (*r* = 0.628, *p* = 7.85 × 10^–9^). However, the viscosity values $\eta$ obtained by SWE were relatively independent of those obtained by DMA with a correlation coefficient of − 0.01. The combined Voigt elasticity measurements have high validity in the prediction of steatosis (S0 vs. S1–S4), with an AUROC of 0.755 (95% CI 0.6175–0.8925, *p* < 0.01) and the optimal cutoff value was 2.08 kPa with a sensitivity of 78% and specificity of 63%.

**Conclusion:**

SWE might have the feasibility to be introduced as an auxiliary technique for NAFLD patients in clinical settings. However, the viscosity results measured by SWE and DMA are significantly different, because the two methods work in different frequency bands.

## Background

Nonalcoholic fatty liver disease (NAFLD) is a main cause of chronic liver disease (CLD) and is rapidly becoming one of the most common liver diseases, with an approximate prevalence of 20–30% of the total population in Western countries [1, 2]. It represents a spectrum of diseases ranging from simple steatosis to nonalcoholic steatohepatitis (NASH), which has a close relationship with liver cirrhosis and hepatocellular carcinoma [3–5]. NASH is critical in the development of liver fibrosis and liver failure and has become a new challenge in the field of liver disease research.

Many different techniques have been studied in the diagnostic of NAFLD. Magnetic resonance elastography (MRE) uses a modified phase-contrast method to image the propagation characteristics of the shear wave in the liver. Early researches of MRE suggest that it could accurately differentiate simple steatosis from NASH with or without fibrosis. However, MRI is costly and time consuming for use in routine clinical practice [6–8]. Recently, several ultrasound-based elastography techniques, including transient elastography (TE) [9–11], acoustic radiation force impulse (ARFI) [12] and shear wave elastography (SWE) [13], have been used for the noninvasive diagnosis of liver disease. Compared with liver biopsy or other imaging methods, ultrasound elastography has the advantages of a lower cost and a smaller time commitment. As the procedure is non-invasive, it can supersede biopsy-related postoperative complications and potential diagnosis error due to a small sampling range [14].

The mechanical properties of liver tissue may be helpful in the diagnosis of liver diseases. Many studies had shown that liver stiffness measured by elastography techniques is strongly linked to liver fibrosis [15, 16]. The World Federation for Ultrasound in Medicine and Biology (WFUMB) recently produced guidelines that recommended the use of elastography techniques for distinguishing advanced fibrosis (≥ F2) from early fibrosis (≤ F1) [17]. However, the mechanism of hepatic steatosis development is different from that of fibrosis. Some researches on steatosis by elastography technology mainly focused on steatosis with fibrosis, while steatosis without fibrosis has been poorly studied. The relationship between steatosis severity and liver stiffness has not been determined [18–20]. Many studies have suggested that liver steatosis with the accumulation of lipid deposits may cause changes in viscoelasticity rather than stiffness. Zhu et al. proposed that the characterization of shear wave dispersion is significantly related to various oil percentages in gelatin phantoms [21]. Barry et al. showed that an increase in liver steatosis increased the viscosity in a mouse liver model, ex vivo [22]. Deffieux et al. [23] found no obvious correlation of liver viscosity and dispersion curve slope with steatosis or disease activity. However, the relationship between liver viscoelasticity and steatosis grade is not clear.

Mechanical tests have been used as another method to assess tissue mechanical properties [24–27]. For example, the dynamic mechanical analysis (DMA) is considered a conventional “gold standard” method to assess the mechanical properties of tissues and can determine the viscoelastic properties of tissues. Many studies have used DMA tests to measure the mechanical properties of liver tissue for comparison of various ultrasound elastography technique results [28, 29].

Our previous study showed the feasibility of assessing the viscoelasticity of liver in a rat model of steatosis by the DMA test [27]. In this study, we used shear wave elastography to evaluate the viscoelasticity of rat liver with steatosis. A DMA test was performed by oscillatory shear test on a liver sample from the same rat model as before, and then parameters, including the elasticity *µ* and the viscosity* η*, were subjected to comparative analysis with variation in the grade of liver steatosis.

## Results

### Histologic characteristics

**Fig. 1:**
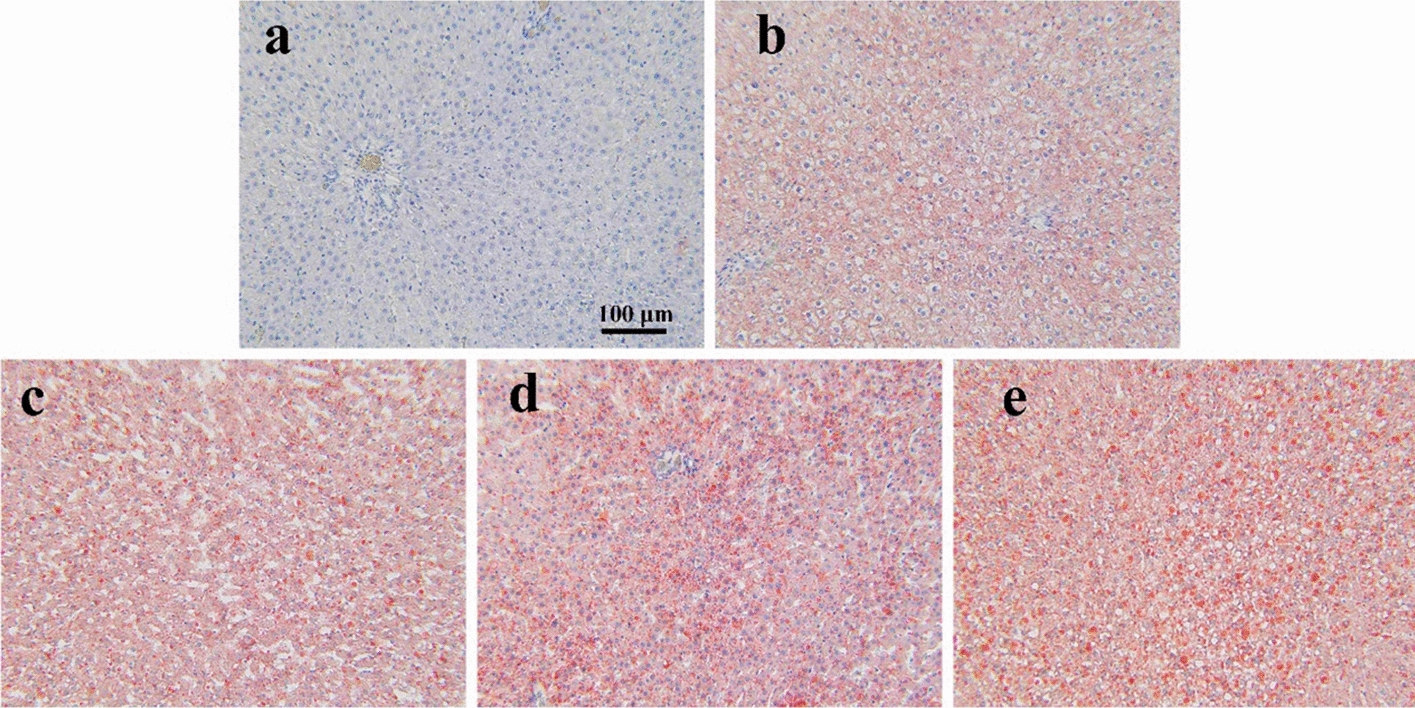
Typical specimens of five rat livers at different liver steatosis stages. **a**–**e** S0–S4

Table 1 lists the distribution of rats with NAFLD according to histologic characteristics. Liver tissue sections from mice in each group after SWE measurements were stained with Oil Red O and examined under the light microscope. Fat droplets in liver tissue were increased significantly as the stages of steatosis increased. Figure 1 shows representative histologic findings according to the severity of NAFLD. Figure 1a illustrates normal histologic findings without steatosis, b and c have certain characteristics in steatosis, d and e show significant hepatocytes containing macrovesicular fat droplets diffused in liver sections after ORO staining, which indicate severe steatosis.

**Table 1:** Number of rats and histopathologic results at each steatosis stage

Days of food administration before sacrificing	Number of rats sacrificed	Steatosis stage determined by the histological assessment of the sacrificed rats				
		S0	S1	S2	S3	S4
Control group	17	17	0	0	0	0
14	12	0	7	4	7	0
28	12	0	3	5	4	0
42	14	0	1	8	5	0
60	14	0	3	2	2	7
Total Number	69	17	14	19	12	7

### Use of SWE and DMA for assessing the grade of steatosis

**Fig. 2:**
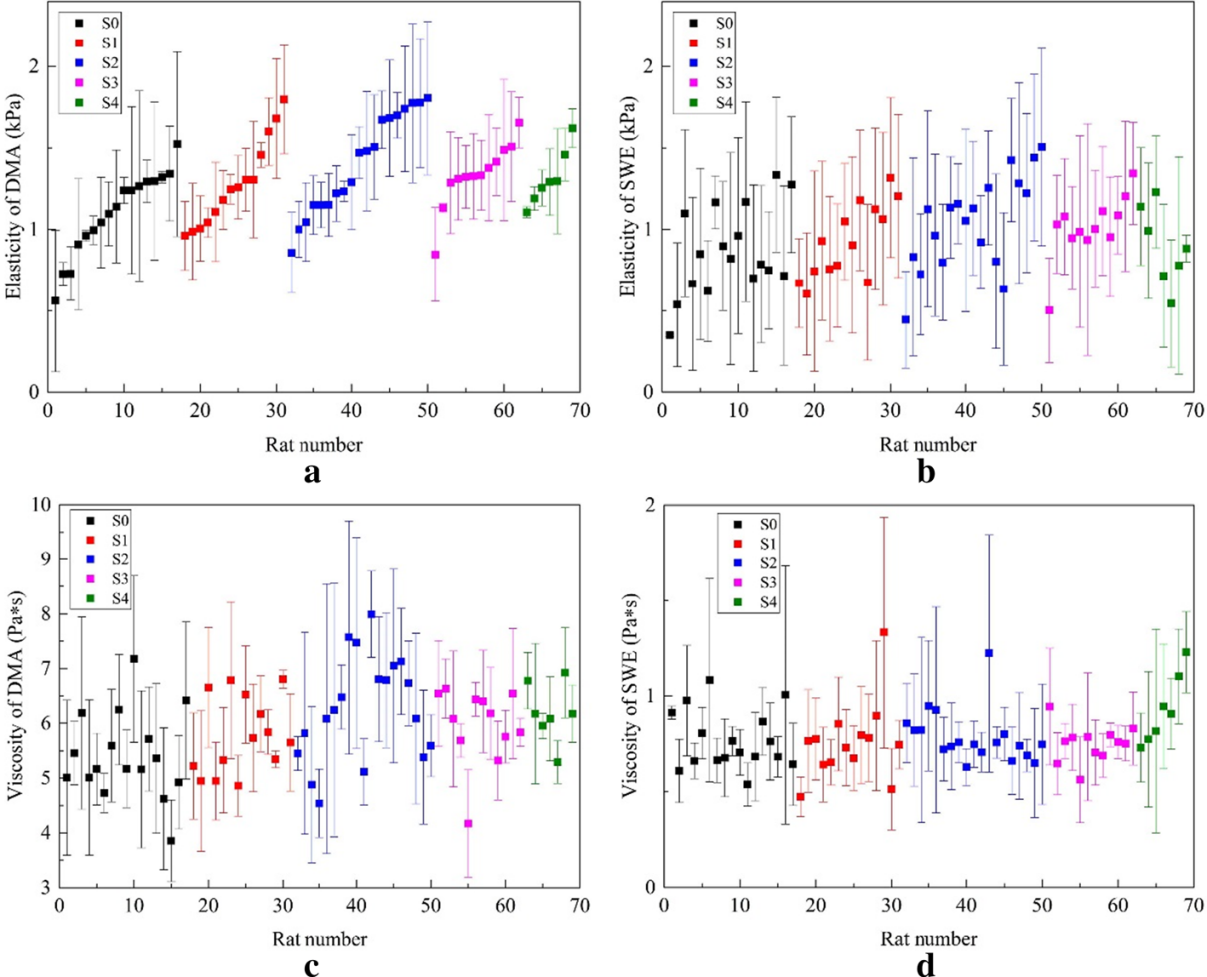
Mean and standard deviation of the elasticity and the viscosity in 69 rats for both methods. For each stage, the rats in this stage were sorted according to their mean elasticity values obtained by the DMA method

The elasticity and viscosity values of 69 rats for S0–S4 obtained by the two methods are shown in Fig. 2. For each stage, the rats in this stage were sorted according to their mean elasticity values obtained by the DMA method. The mean elasticity and viscosity values for each stage are listed in Table 2. The mean values of *µ* obtained by SWE (from 0.86 ± 0.27 kPa to 1.05 ± 0.29 kPa) were slightly lower than those by DMA (from 1.12 ± 0.21 kPa to 1.39 ± 0.31 kPa). For viscosity, the values of DMA (from 5.40 ± 0.78 Pa*s to 6.27 ± 0.96 Pa*s) were significant greater than those of SWE (from 0.75 ± 0.10 Pa*s to 0.92 ± 0.18 Pa*s). The *p* value for each of *µ* and *η*, between the two types of measurements in each degree of steatosis, all less than 0.05.

**Table 2:** Mean viscoelasticity parameters of all rat livers measured by SWE and DMA methods

Degree of liver	*n*	SWE		DMA	
Steatosis		Elasticity (kPa)	Viscosity (Pa*s)	Elasticity (kPa)	Viscosity (Pa*s)
S0	17	0.86 ± 0.27(*)	0.77 ± 0.15(**)	1.12 ± 0.21	5.40 ± 0.78
S1	14	0.93 ± 0.23(**)	0.76 ± 0.20(**)	1.25 ± 0.26	5.77 ± 0.71
S2	19	1.05 ± 0.29(**)	0.79 ± 0.14(**)	1.39 ± 0.31	6.27 ± 0.96
S3	12	1.01 ± 0.20(**)	0.75 ± 0.10(**)	1.29 ± 0.18	5.97 ± 0.68
S4	7	0.90 ± 0.24(**)	0.92 ± 0.18(**)	1.31 ± 0.14	6.20 ± 0.54

*n* represents the number of rats. In SWE, 10 points in each rat liver were measured in vivo, and 3 liver samples were extracted in random from each rat in DMA, values are the mean ± standard deviation. () represents the *p* value for each of $\mu$ and$\eta$, between the two types of measurements in each degree of steatosis. *p < 0.05, **p < 0.01.

**Fig. 3:**
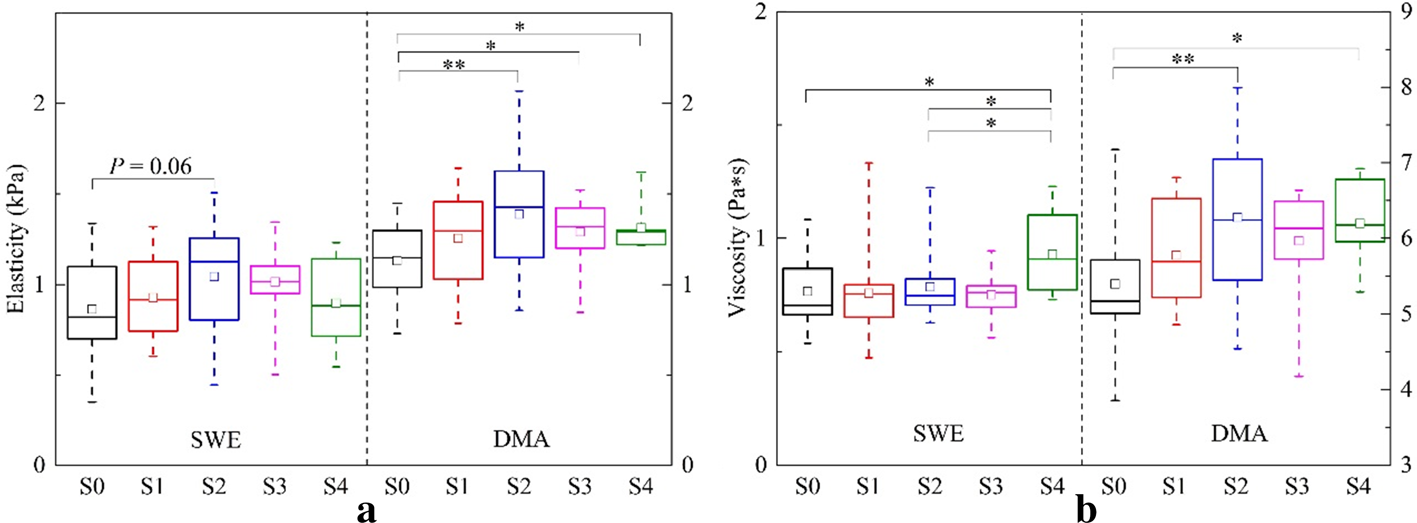
Boxplot of **a** elasticity and **b** viscosity obtained by the two methods at five steatosis stages. The upper and lower boundaries of the boxes represent the 25th and 75th percentiles, lines within the boxes represent medians, squares within the boxes represent means, and error bars represent ranges. *Asterisks* indicate the pairs having statistically significant differences in the Tukey–Kramer test after the ANOVA test. *p < 0.05, **p < 0.01

The box plots in Fig. 3 illustrate the variations of elasticity and viscosity of the two methods for each stage. The results of the ANOVA test with Tukey–Kramer multiple comparison tests found out the group pairs which had significant differences in elasticity or viscosity.

### Correlation of SWE and DMA on the elasticity and the viscosity

**Fig. 4:**
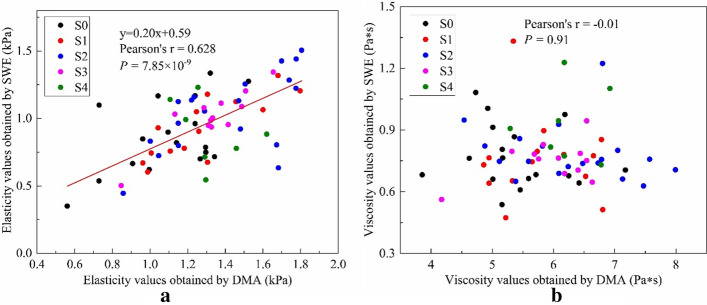
Correlative analysis between the SWE and DMA about **a** elasticity and **b** viscosity at five steatosis stages

Correlations between the SWE and DMA for the elasticity and viscosity are illustrated in Fig. 4. The Pearson’s correlation coefficients indicate that elasticity obtained by SWE is positively linear correlated with DMA (*r* = 0.628, *p* = 7.85 × 10^–9^), while there is almost no correlation between viscosity obtained by SWE and DMA (*r* =  − 0.01, *p* =  − 0.91).

### Combined Voigt model analysis

**Fig. 5:**
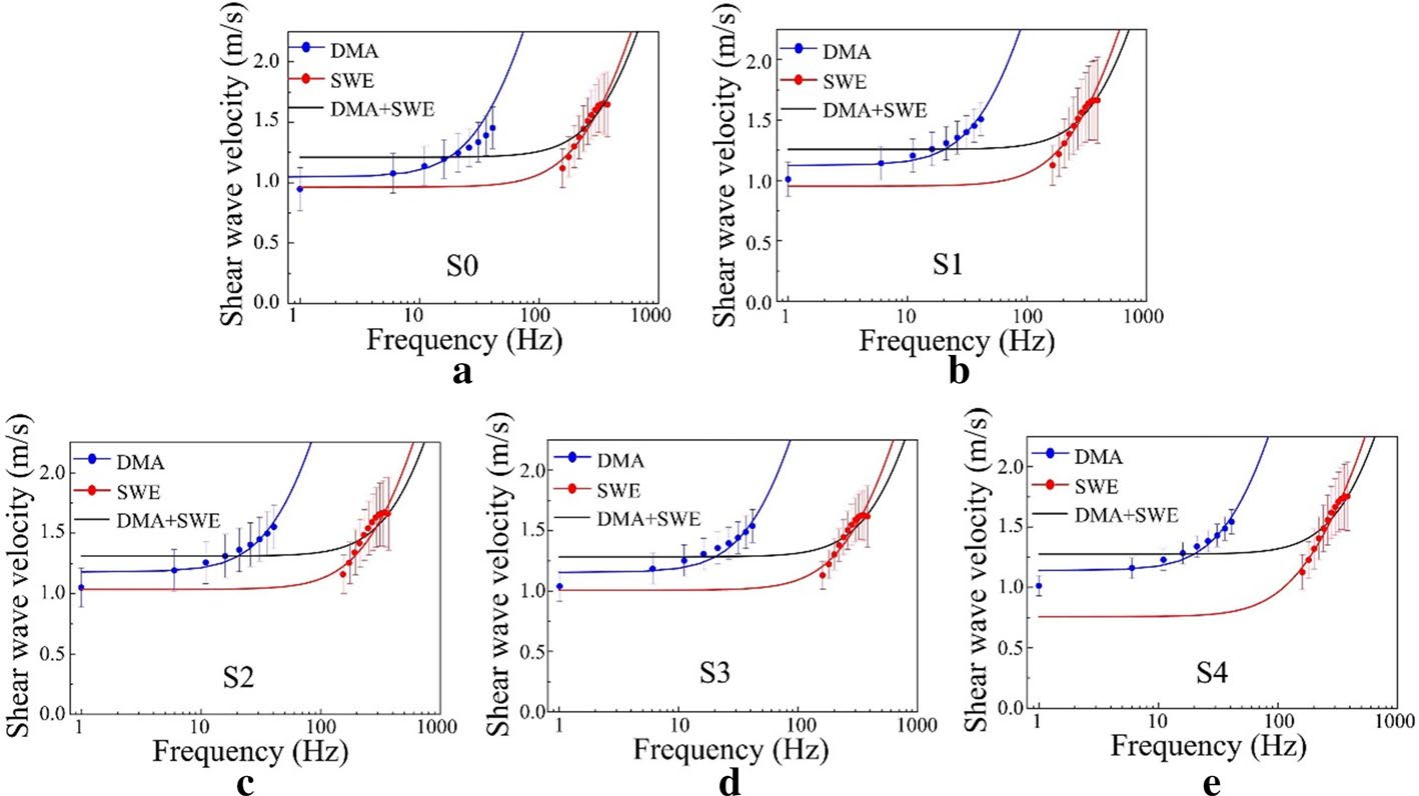
Fitting curves for the DMA data (blue line), the SWE data (red line), and the combination of the DMA and SWE data at different liver steatosis stages (**a**–**e**) S0–S4. The circles are the mean values of the phase velocity over the 69 rats and the horizontal bars are the standard deviations

**Fig. 6:**
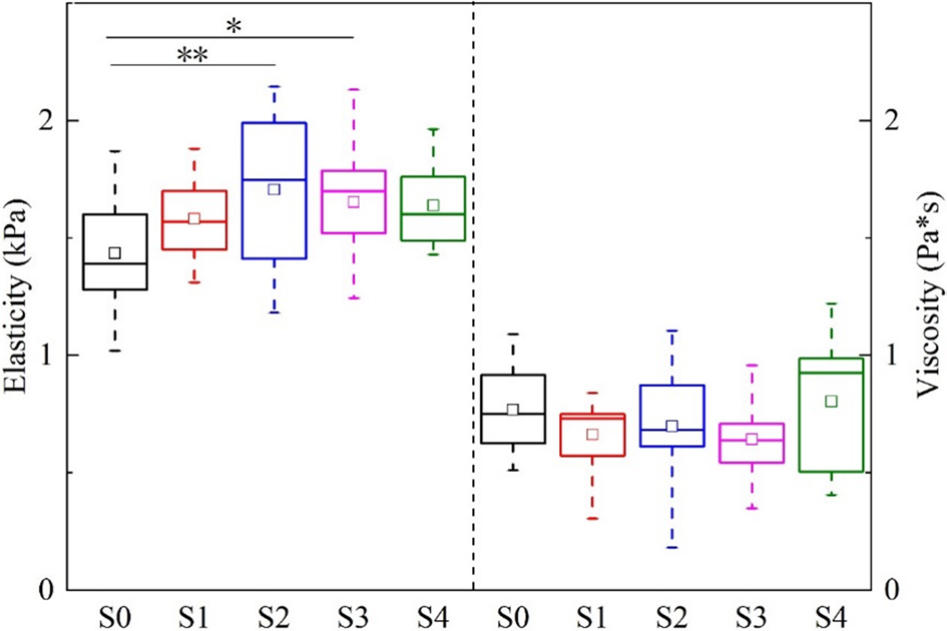
Box plots of elasticity and viscosity estimates in various degrees of steatotic severity from the combined Voigt model analysis

As shown in Fig. 5. The black curves a–e represent combined Voigt model analysis data at each steatosis stage, which are fitted by combined low and high frequency band (1–380 Hz) from DMA and SWE measurements. Figure 6 shows box plots of elasticity and viscosity estimates in various degrees of steatotic severity from the combined Voigt model analysis. Liver elasticity of S3 group (mean ± SD, 1.65 ± 0.23) was significantly higher than that of the S0 group (mean ± SD, 1.43 ± 0.22) (*p* < 0.05). A significant difference was found between the S2 and S0 groups in combined elasticity (*p* < 0.01), but no significant difference was observed between the other groups.

**Fig. 7:**
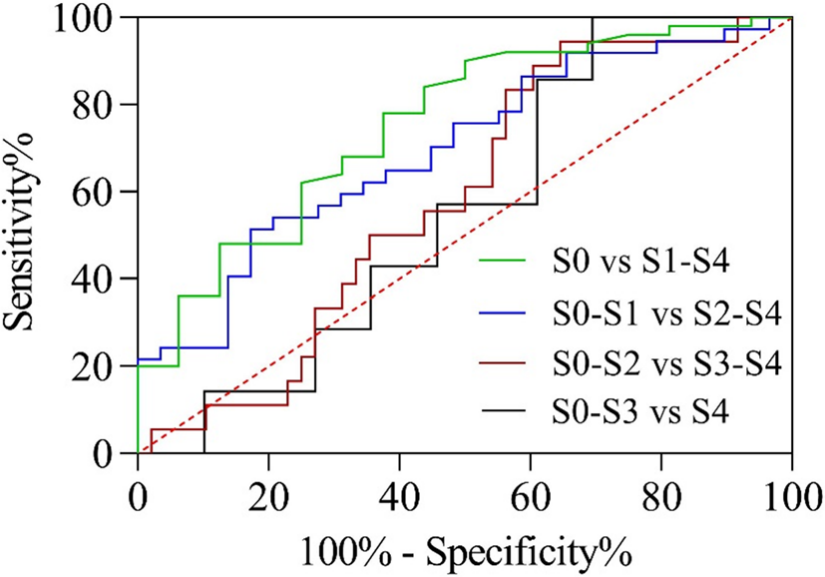
Receiver operating characteristic curves based on combined Voigt elasticity for differentiation of different steatosis grades by combined Voigt model analysis

The performance of the viscoelastic parameters in grading the steatosis stages S0–S4 is studied using the receiver operating characteristic (ROC) curves (Fig. 7), these curves represent sensitivity vs. 1-specificity for all possible cutoff values for the prediction of the different stages. The discriminating cutoff values, which are chosen by maximizing the Youden index for the ROC curves, sensitivity and specificity values are computed with exact 95% confidence intervals. Figure 7 shows the combined Voigt elasticity measurements have high validity in the prediction of steatosis (S0 vs. S1–S4), with an AUROC of 0.755 (95% CI 0.6175–0.8925, *p* < 0.01) and the optimal cutoff value was 2.08 kPa with a sensitivity of 78% and specificity of 63%. Combined Voigt model analysis was also able to grade steatosis severity, S ≥ 2 (AUROC = 0.697, 95% CI 0.5707–0.8235, *p* < 0.01, optimal cutoff value was 2.98 kPa with a sensitivity of 51% and specificity of 83%).

## Discussion

NAFLD is known to be a complex disorder and includes simple steatosis, NASH, and fibrosis. In clinical situations, steatosis and fibrosis are usually interleaved together, and it is difficult to distinguish their separate effects on the mechanical properties of the liver tissue. Many clinical researchers of elastography technology have mainly focused on steatosis with fibrosis or NASH. Grimal et al. [30] investigated the relationship between liver tissue stiffness and histological inflammation score, hepatic fibrosis stage, ballooning score, steatosis analyzed by SWE in rats with NASH, and found that median liver stiffness values measured using SWE showed a stepwise increase with increasing steatosis grade (*p* = 0.03), the results were consistent with those of [31] that demonstrated that liver elasticity was effective in detecting NAFLD. However, Nightingale et al. [32] performed shear wave dispersion with a linear dispersion model to perform shear wave dispersion analysis in traditionally “difficult-to-image” subjects in 135 NAFLD patients. Nevertheless, viscoelastic parameters were not related to the steatosis stage. The presence of entirely different results in the relationship between liver viscoelasticity and steatosis stage may have many causal factors, such as different frequency ranges corresponding to different viscoelastic parameters, experimental conditions, machines, and operators. Moreover, the role of SWE measurement in vivo is more complicated because of the complex structure of the organism and variable factors. It also becomes difficult to evaluate when a patient has more than just a single disease in the clinical setting. Parker et al. [33] summarized numerous studies about ultrasound shear wave dispersion in healthy and steatosis livers and concluded that animal models and human populations need further investigation.

In this study, we used a rat model of liver steatosis, which avoided the confounder of liver fibrosis, thus we can focus on studying the effect of steatosis on viscoelasticity. Disease development in the soft tissue is always accompanied by a variety of mechanical properties of the tissues. The viscoelastic properties of tissue are promising for utilizing the inherent mechanical properties of tissue for grading disease. To this end, we performed liver viscoelasticity measurements using SWE in rat models with various degrees of steatosis severity. The DMA tests were used as a standard of reference, and the values of elasticity and viscosity obtained by SWE were compared to the degree of steatosis as evaluated by histological assessment. In this study, we used the Voigt model to obtain the viscoelasticity parameters in a rat model of NAFLD. We found that the values of elasticity obtained by SWE increased from stage S0 to S2 (Fig. 3a), which was similar to the trend of DMA. Interestingly, the values decrease slightly from stage S2 to S4, which is also similar to the DMA. Although the trend between the values of elasticity obtained by SWE is consistent with DMA, the *p* values of elasticity obtained by SWE were slightly greater than 0.05 and not statistically different among the five steatosis stages. The elasticity of DMA was statistically different between stages S0 and S2–S4. The values of elasticity obtained by SWE were relatively lower than those obtained by DMA. However, compared with the viscosity of DMA, the results of SWE were significantly lower, and there was no difference from S0 to S3. The mean value of S4 was higher than in other stages (Fig. 3b). The trends of the five stages of viscosity obtained by SWE were not consistent with those obtained by DMA [21]. Although some phantom studies have shown that viscosity can provide relevant and independent information about the inherent state, until now, few researchers could determine the viscoelasticity parameters in vivo for the diagnosis of fatty liver disease.

Despite the results obtained by SWE based on shear wave dispersion, accurate staging of the grade of liver steatosis did not prove statistically significant. However, it is interesting to evaluate the values of viscoelasticity relationships between SWE and DMA. In Fig. 4, the mean values of SWE were moderately correlated with those obtained by DMA; the correlation coefficient was 0.628, and *p* = 7.85 × 10^–9^, indicating significant linear correlation. However, the mean values of viscosity measurements obtained by SWE were relatively independent of those obtained by DMA with a correlation coefficient of − 0.01. This result suggests that the elasticity may provide useful information about the liver steatosis status obtained by SWE. There is little relationship between SWE and DMA in terms of viscosity, suggesting that it has a weak correlation with liver steatosis, which is consistent with previously reported results [23].

The viscosity results measured by the two methods are significantly different, because the measurements depend heavily on the frequency bands. It is focused to investigate why the elasticity values measured by SWE have the same trend with the DMA but without a statistical difference among the five steatosis stages. There may be several reasons for this result. First, DMA has much lower frequencies than SWE, which may lead to different vibrations in microstructures of liver tissue. Also, the frequency ranges of the shear wave measured using SWE may not reveal the actual viscoelastic properties of the tissue [34]. In DMA tests, the frequency ranged from 1 to 41 Hz, while the shear wave frequency of SWE ranged from 160 to 380 Hz. Specifically, the excised liver has no blood circulation in the DMA tests. Blood flow can affect the viscoelasticity measurement of the liver by SWE [35]. Also, the limited size of the species utilized and ultrasound imaging quality considerations can affect the results.

According to Eq. (1), the elasticity parameters are dependent on the low-frequency shear wave velocity. The elasticity of DMA was statistically different between stages S0 and S2–S4. In this study we took a step further  using combined Voigt model analysis, which combined low and high frequency band (1–380 Hz) from DMA and SWE measurements, then the combined elasticity and viscosity were derived from Voigt model. Figure 5 shows that the means of the shear wave velocities at frequencies from 1 to 380 Hz increased with increasing frequency. The results shown in the box plots (Fig. 6) and the ROC curves (Fig. 7) indicate that the combined elasticity was statistically significantly different between stage S ≥ 1 and stage S ≥ 2. The AUROC values for combined elasticity for distinguishing liver steatosis stages (S ≥ 1) were approximately 0.755. These results find coincide with a previous study by Nightingale et al. [32], they found that the AUROC values for distinguishing the liver steatosis stages (S ≥ 2) were 0.49 for the elasticity at 200 Hz. Although it seems reasonable that the accumulation of fat may result in changes in the liver viscosity parameters, the viscosity value could not be used to evaluate the level of liver steatosis. The results can be attributed to multiple causes, some of them limited frequencies of the induced shear wave, because the viscosity becomes prominent at high frequencies. However, the shear wave is difficult to detect at high frequencies because of the high level of shear wave attenuation.

## Conclusion

In this study, we compared the measurement of viscoelastic parameters, including elasticity and viscosity, by DMA tests, SWE methods and combined Voigt model analysis in a rat model of NAFLD at five steatosis stages. The results showed that the elasticity values from the two methods followed the same trend, suggesting that elasticity *µ* has a significant correlation with liver steatosis. To some extent, elasticity values measured by SWE can reflect liver steatosis processes. SWE might have the feasibility to be introduced as an auxiliary technique for NAFLD patients in clinical settings.

## Materials and methods

### Animal model

A total of 69 male Sprague–Dawley (SD) rats (weighing 200 ± 20 g, Guangdong Medical Laboratory Animal Center, Foshan, China) were used in this study. They were maintained under Specific Pathogen Free (SPF) conditions (relatively constant temperature: 20–26 °C humidity: 30–50%, 10 h: 14 h light/dark rhythm alternately) with a high-calorie diet. To induce hepatic steatosis in different stages (S1–S4), a high-calorie diet for different numbers of days were supplied to SD rats at a dose of 1 mL/100 g rat weight once a day for 60 days. The control group (S0) was fed with a regular diet. Rat livers were harvested for DMA tests and histologic assessment after the rat had been used for in vivo ultrasound measurements. The histopathologic examination ultimately determined the hepatic steatosis stage. The number of animals in each group is stated in Table 1. Animal care and experiments were approved by the Animal Care and Use Committee of School of Medicine in Shenzhen University and Guangdong Medical Laboratory Animal Center.

### In vivo SWE measurement

**Fig. 8:**
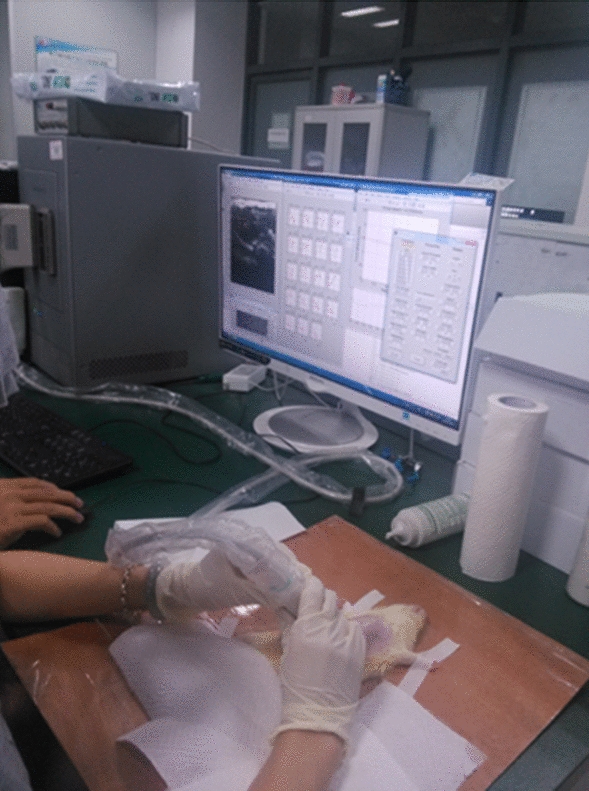
Experimental setup for in vivo shear wave elastography (SWE) measurement

In this experiment, the rat was placed in the supine position on the experimental table after anesthesia at a relatively constant temperature of 24–26 °C. The rat abdomen was shaved and coated with ultrasonic gel, and then an ultrasound B-mode image was used for localization of the liver and a region of interest (ROI) (size: 5 mm × 15 mm) in the median lobe of the liver, devoid of large vessels, was chosen for measurement. After the ROI of the liver was selected, the operator kept the probe stable and switched to the SWE mode to finish the measurement, as shown in Fig. 8. To obtain a relatively accurate result, for each rat, measurements were repeated ten times in ten locations, and the calculated elasticity *µ* and viscosity *η* were the mean of ten valid measurements.

The SWE measurement was performed on the Verasonic vantage 256 systems (Verasonics Inc., Kirkland, WA, USA) with a linear array transducer L11-4v (Philips Healthcare, Andover, MA). The measurement procedure is similar to that used in the previous study [36]. First, a B mode image of the liver was obtained to provide imaging guidance for selecting the measurement locations, then a region of interest (ROI), 5 mm axially × 15 mm laterally, was selected for measurement. A total of 20 array elements were used in one excitation, which consisted of three successive push beams to create a Mach cone [37]. These three push beams were focused at − 5 mm, 0 mm, and 5 mm deep relative to the center of the ROI with a time interval of 50 microseconds. The push beam at each depth was generated by an 80-microsecond tone burst at a center frequency of 4.4 MHz. Second, the system was switched immediately to the plane wave imaging mode after the push wave transmission. All elements were set to fire 50 detect pulses with a center frequency of 6.25 MHz and a pulse repetition frequency of 10 kHz. Next, the data post-processing steps include: (1) In-phase quadrature (IQ) data within the ROI was processed with a 2D correlation algorithm to obtain particle velocity [38]; (2) 10 pixels spatial averaging was applied in the axial direction to remove spike noise after the 3 × 3 median filter, cubic spline interpolation with a factor of five was conducted to reconstruct the displacement profiles to increase the temporal resolution; (3) low-pass filtering with a cut off frequency of 1000 Hz was used to reduce jitter after the reverberation frames were removed, then a Fourier transform was used to estimate the phase at each frequency; (4) then a directional filter was applied to reduce the artifacts from reflected shear waves at boundaries, a linear regression algorithm was used to calculate the shear wave phase velocities at each frequency (160–380 Hz) by estimating the average phase difference [39]; (5) In this study, it is assumed that the liver consists of viscoelastic and isotropic homogenous material. The relationship between the shear wave velocity and frequency was derived from the Voigt model. The equation is as shown below [40]:

1 $$c_{s} (\omega ) = \sqrt {\frac{{2(\mu^{2} + \omega^{2} \eta^{2} )}}{{\rho (\mu + \sqrt {\mu^{2} + \omega^{2} \eta^{2} } )}}}$$

where $c_{s}$ is the shear velocity, $\omega$ the angular frequency, $\rho$ the mass density, $\mu$ and $\eta$ are the shear elasticity and shear viscosity. Finally *µ* and *η* were estimated by MATLAB (Mathworks, R2014a) with a nonlinear fitting method using Eq. 1 based on a least-squares criterion.

### Ex vivo DMA experiment

The principle of DMA is performed as previously described [27]. Briefly, for homogeneous biological tissue, the sinusoidal shear strain $\varepsilon (t) = \varepsilon_{0} e^{j\omega t}$ induces a corresponding sinusoidal stress$\sigma(t)=\sigma_0 e^{j(\omega t + \delta)}$. The complex shear modulus $G^{*}$ is defined as the ratio of stress to the strain:

2 $$G^{*}(\omega)=\frac{\sigma(t)}{\varepsilon(t)}=\frac{\sigma_0 e^{j(\omega t + \delta)}}{\varepsilon_0 e^{jwt}}=\frac{\sigma_0}{\varepsilon_0}(\text{cos} \delta + j\, \text{sin} \delta)$$

where $\omega$ is the angular frequency, $\delta$ is a phase difference, $\sigma_{0}$ and $\varepsilon_{0}$ are the shear stress amplitude and strain amplitude, respectively. The real part of Eq. 2, $G^{^{\prime}} (\omega )$ is the storage modulus relating to the elasticity *µ*, while the imaginary part $G^{\prime \prime} (\omega )$ is the loss modulus relating to the viscosity *η*. Firstly, the frequency and amplitude of stress $F^{*}$ are set by DMA system, the engineering strain $X^{*}$ corresponding to the engineering stress response is obtained by sensor measurement. So the phase delay δ can be estimated. The results of shear mode measurement are adjusted by the shape of the sample (Width*Length*Thickness = 5 mm × 15 mm × 4 mm, the cross sectional area is 5 mm × 15 mm).

3 $$G^{\prime}=\left(\frac{F^{*}}{X^{*}} \text{cos} \, \delta \right)/\left( \frac{Width * Length}{Thickness}\right)$$

4 $$G^{\prime \prime}=\left(\frac{F^{*}}{X^{*}} \text{cos} \, \delta \right)/\left( \frac{Width * Length}{Thickness}\right)$$

For a linear viscoelastic tissue, the relationship between the shear wave velocity $c(\omega)$ and the complex shear modulus $G^{*}$ is as follows:

5 $$c(\omega)=\sqrt{\frac{\vert G^{*} (\omega) \vert}{\rho}}$$

where $\rho$ is the density of tissue. The shear wave phase velocity can be calculated by substituting Eq. 3 and Eq. 4 into Eq. 5. The relationship between the phase velocity and the complex modulus is expressed as Eq. 6.

6 $$c(\omega)=\sqrt{\frac{2(G^{{\prime}2}+G^{{\prime\prime}2})}{\rho G^{\prime}\left(1+{\left(\frac{G^{\prime\prime}}{G^{\prime}}\right)}^2\right)}}=\sqrt{\frac{2(G^{{\prime}2}+G^{{\prime\prime}2})}{\rho (G^{\prime}+\sqrt{G^{{\prime}2}+G^{{\prime\prime}2}})}}$$

Various rheological models have been investigated to describe the tissue, such as the Maxwell, Zener, and Voigt models. Among them, the Voigt model is one of the most commonly used, especially in the studies for assessing liver viscoelasticity [41]. Moreover, a pre-study of rheological experiments were carried out to quantify the mechanical behavior of rat livers at different steatosis stages. The Voigt, Maxwell, and Zener models were used to analyze the mechanical properties of each steatosis stage, the model should not only be satisfied with the DMA experiment of low frequency (1–41 Hz), but also the SWE experiment of high frequency (160–380 Hz). Therefore, the fitness of the three models to shear wave velocity are summarized in Table 3.

**Table 3:** Fitting effect of the three models (n represents the number of rats or samples)

Model	Determination coefficient *R*^2^			
	SWE		DMA	
	Normal (*n* = 3)	Steatosis (*n* = 3)	Normal (*n* = 6)	Steatosis (*n* = 6)
Voigt	0.80	0.84	0.89	0.88
Maxwell	0.35	0.35	0.52	0.53
Zener	0.54	0.44	0.94	0.92

As shown in the table above, the results revealed that the Voigt model is the optimal model for describing the mechanical properties of each steatosis stage in rats. To quantitatively analyze the viscoelastic properties of the tissues, the phase velocity can be expressed by Eq. 6, Therefore, the elasticity and viscosity properties can be estimated from the shear wave velocity dispersion curve according to the Voigt model (Eq. 1 [42]).

Each rat liver was harvested immediately for ex vivo DMA test after the in vivo ultrasound measurement. The livers were soaked with saline solution to prevent desiccation of liver specimens and kept in the refrigerator at a temperature of 4 °C to preserve freshness.

**Fig. 9:**
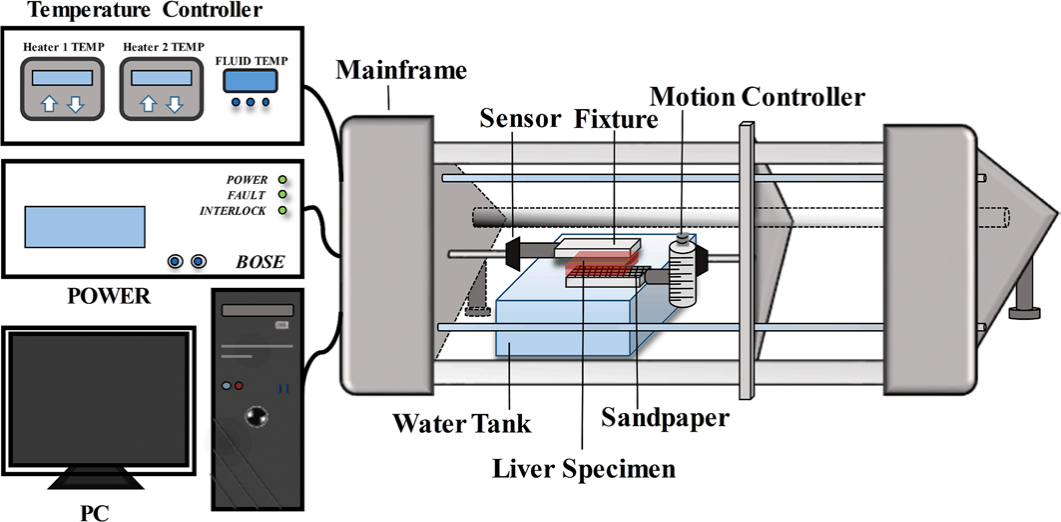
Dynamic mechanical analysis (DMA) experimental platform

Figure 9 shows the DMA experimental instrument (the ElectroForce3200 Series, Bose Corp., Eden Prairie, Minnesota, USA) operating at a parallel plate shear mode. Two to three liver specimens were taken from each rat, and then each specimen was cut by a scalpel into the same shape (size: 15 mm × 5 mm × 4 mm, thickness: 4 ± 1 mm) to match the parallel plates. During the experiment, the liver specimen was fixed to the fixture in the water tank which was filled with a saline solution at a body temperature of 38.1 ± 0.2 °C. The sandpaper was attached between the parallel plates to prevent the liver specimen from slipping. The motion controller was rotated slowly to ensure sufficient contact between the parallel plates. After the sample was placed on the parallel plates, the sample slowly received a precompression of 2–3 g to ensure sufficient contact between the plate and the sample and avoid slippage. Strain sweeping oscillation tests were performed to ensure that the strain and the frequency were within a reasonable range. A strain of 1% at 1–41 Hz was used for the subsequent frequency sweeping oscillations tests.

A sinusoidal torque was engendered by the machine to make the liver specimen generate stress and strain. Then, the dynamic mechanical properties, including storage modulus $G^{^{\prime}}$ and loss modulus $G^{^{\prime\prime}}$ were directly obtained from the system during the test. The phase velocities at all frequency points in the range of 1–41 Hz with an interval of 5 Hz were substituted in Eq. 3 and Eq. 4, and then shear elasticity $\mu$ and shear viscosity $\eta$ of every sample were estimated by non-linear curve-fitting.

### Combined Voigt model

However, DMA is better than SWE in pathological diagnosis, the main reason is that DMA technology is the gold standard for the measurement of mechanical properties of materials. The storage modulus $G^{^{\prime}}$ and loss modulus $G^{^{\prime\prime}}$ can be obtained by directly applying stress or strain to the liver. Therefore, the elasticity and viscosity properties can be estimated from the shear wave velocity dispersion curve according to Eq. 6. SWE technology is to obtain the mechanical properties of tissue by ultrasonic indirect detection, many external factors may affect the ultrasonic detection. From the perspective of model fitting, the shear wave velocity fitting with a wide range of frequencies can more accurately reflect the mechanical properties of the tissue. In this experiment, the scanning frequency is 1–41 Hz. The data will be distorted when the frequency is higher than 41 Hz, which is mainly due to the limitation of the mechanical properties of liver tissue itself. To go further, a combined Voigt model analysis was used to fit phase velocity–frequency data, including the means and standard deviations of the phase velocities for frequencies range from 1 to 41 Hz (DMA), 160–380 Hz (SWE), and 1–380 Hz (DMA + SWE) at each steatosis stage.

### Histological assessment

The excised liver tissues from middle part of the left lobe were fixed in the 10% neutral formalin liquid, and conventional methods of dehydration, paraffin-embedded sections, and Oil Red O staining was conducted by histopathology technicians in the Guangdong Medical Laboratory Animal Center. Two slices, with a thickness of 7 μm, from each rat were used for the histologic examination. These pathological slices were observed under an optical microscope (BX41, Olympus, Pittsburgh, PA, USA) by pathologists (experience of 20 y) who was blinded to the different treatment groups and data from ultrasound measurements. Stages of steatosis were determined by quantifying percentages of hepatocellular macrovesicular steatosis: 0–4% (S0); 5–25% (S1); 26–50% (S2); 51–75% (S3); > 76% (S4). The percentage was calculated by the counting software in the microscope. Histologic analysis was performed according to the scoring system developed by Kleiner’s group [43]. Figure 1 shows typical liver specimens for the five steatosis stages.

### Statistical analysis

All 69 rats were divided into five groups separated by histological grade (S0–S4). For each steatosis grade, the viscoelasticity parameter values of all the rats at that grade were grouped. The mean parameter value of each rat was then used to estimate the population mean of each steatosis group. If the value exceeded more than 1.5 times the interquartile range in its group, it was eliminated as an outlier and the corresponding measurement was excluded [27]. The relationship between the SWE and DMA on *µ* and $\eta$ was analyzed using the Pearson correlation coefficient and linear regression. The accuracy of the distinction by combined dispersion curve analysis for grading steatosis severity was assessed using a nonparametric area under receiver operating characteristic curve (AUROC), and the corresponding sensitivities and specificities were calculated at optimal cut-off values of liver elasticity *µ*. All data were represented as means ± standard deviation (SD) and analyzed using the two-tailed Student *t* test, analysis of variance. The one-way analysis of variance (ANOVA) with the Tukey–Kramer multiple comparison tests were used to compare the liver viscoelasticity parameters among the steatosis stages. Differences were considered significant when *p < 0.05, **p < 0.01, and ***p < 0.001, respectively.

## Data Availability

The data used to support the findings of this study are available from the corresponding author upon reasonable request.

## Abbreviations

NAFLD: Nonalcoholic fatty liver disease
NASH: Non-alcoholic steatohepatitis
SWE: Shear wave elastography
DMA: Dynamic mechanical analysis
MRE: Magnetic resonance elastography
TE: Transient elastography
ARFI: Acoustic radiation force impulse
SD: Sprague–Dawley
SPF: Specific Pathogen Free
ROI: Region of interest
IQ: In-phase quadrature
ANOVA: Analysis of variance
